# Spectro-temporal acoustic elements of music interact in an integrated way to modulate emotional responses in pigs

**DOI:** 10.1038/s41598-023-30057-5

**Published:** 2023-02-21

**Authors:** Juliana Zapata Cardona, Maria Camila Ceballos, Ariel Marcel Tarazona Morales, Edimer David Jaramillo, Berardo de Jesús Rodríguez

**Affiliations:** 1 Grupo de Investigación en Patobiología QUIRON, Escuela de Medicina Veterinaria, Universidad de Antioquia, Calle 70 No. 52-21, Medellín, Colombia; 2 Faculty of Veterinary Medicine, University of Calgary, Clinical Skills Building, 11877-85th Street NW, Calgary, AB T3R 1J3 Canada; 3 Grupo de Investigación BIOGEM, Facultad de Ciencias Agrarias, Universidad Nacional de Colombia, Sede Medellín, Cra. 65 No. 59A-110, Medellín, Colombia; 4 Grupo de Investigación Nutri-Solla, Solla S.A., Carrera 42 # 33-80, Itagüí, Colombia

**Keywords:** Animal behaviour, Coevolution

## Abstract

Music is a complex stimulus, with various spectro-temporal acoustic elements determining one of the most important attributes of music, the ability to elicit emotions. Effects of various musical acoustic elements on emotions in non-human animals have not been studied with an integrated approach. However, this knowledge is important to design music to provide environmental enrichment for non-human species. Thirty-nine instrumental musical pieces were composed and used to determine effects of various acoustic parameters on emotional responses in farm pigs. Video recordings (n = 50) of pigs in the nursery phase (7–9 week old) were gathered and emotional responses induced by stimuli were evaluated with Qualitative Behavioral Assessment (QBA). Non-parametric statistical models (Generalized Additive Models, Decision Trees, Random Forests, and XGBoost) were applied and compared to evaluate relationships between acoustic parameters and pigs’ observed emotional responses. We concluded that musical structure affected emotional responses of pigs. The valence of modulated emotions depended on integrated and simultaneous interactions of various spectral and temporal structural components of music that can be readily modified. This new knowledge supports design of musical stimuli to be used as environmental enrichment for non-human animals.

## Introduction

Music has various spectral and temporal structural elements. In humans, interactions of these components determine the emotional content of music^1–3^. Furthermore, these properties can be altered to reliably influence emotional valence^4^. In any musical piece, various structural components are simultaneously present and their perception is a holistic process^5^. Perhaps characteristics of music that are effective in inducing and communicating emotional responses in humans could also be applied to other species^6^.

The strong association between music and emotions derives from neurocognitive processes^7,8^. Musical aspects such as tone, rhythm, timbre, frequency, harmony, and melody have been associated with activity in various brain areas, some of which are related to emotions processing and arousal control systems, e.g., those responsible for release of norepinephrine and serotonin, substances involved in regulation of emotional responses^9–13^. Mammals and birds have neuroanatomical structures enabling neurocognitive processing of musical components^14^. Additionally, music is an emotional communication tool encoded within ancient neural circuits, many of which are homologous between humans and other animals^7^. Such perceptions can explain why animals express emotional changes when exposed to musical stimuli^15^.

Music structure evaluation can be performed quantitatively, based on acoustics and musical informatics. This widely used evaluation, also known as digital music analysis^16^, enables extraction of numerical characteristics suitable for statistical analysis^17^. In humans, evaluation of emotions based on acoustic characteristics has been widely developed^18,19^. However, it is common to separately evaluate spectral or temporal structures; therefore, the interaction between these musical elements and their relationship with emotional effects remains largely unknown.

In nonhuman animals, there is a lack of studies evaluating effects of music structure. Some studies evaluated some musical aspects, e.g., type of musical instrument, musical rhythm and harmony, but considered musical parameters separately, and only assessed effects on animals’ behavioral responses^20–22^. To our knowledge, only one study has evaluated effects of one music parameter (harmonic characteristics) on animal’s emotions^15^. Therefore, there is a paucity of studies evaluating effects of various acoustic characteristics on animals’ emotional responses. This approach may be especially relevant in non-human species with specific communication systems and auditory characteristics, including particular auditory ranges of frequency, timing, and other acoustic features necessary for proper music encoding and neurocognitive processing. An acoustic stimulus is effective if it is appropriate for the sensory and communication systems of the species under study^6^. Consequently, species-specific adjustments are essential to develop acoustic stimuli useful as environmental enrichment^6,23^.

Regarding psychoacoustics, swine have an auditory sensitivity similar to primates^24,25^. The auditory range of pigs (40.5 Hz to 40 kHz) is closer to humans (20 Hz to 20 kHz) than to other commonly used animal models, such as mice (1 Hz to 90 kHz) and rats (250 Hz to 64 kHz) whose auditory perception is in the ultrasonic range^26,27^. These characteristics provide a great translational value for using swine for comparative studies with humans and make them a useful model to study music.

Previous research by our team demonstrated that music modulates emotion in pigs and evaluated effects of music as a relevant biological signal, distinguishing its effects from control conditions such as silence^15^. The objective of this study was to perform temporal-spectral analyses of music characteristics, using a music informatics approach, to evaluate effects on emotional responses in farm pigs. We integrated animals’ emotional responses evaluation from a psychoacoustic approach that is useful for investigating potential effects of auditory stimulation in animals and will contribute to designing and refining musical stimuli for environmental enrichment in non-human animals.

## Methods

### Ethical considerations

Experiments were conducted in accordance with ARRIVE guidelines (https://arriveguidelines.org), and all methods were performed following current regulations. The Ethics Committee on Animal Experimentation of the Universidad de Antioquia (CEEA—Act No. 16, April 10, 2018) approved all procedures.

### Study location

This study was performed at Universidad of Antioquia pig farm (6°26′59.606 N 75°32′37.088 W BH-Mb), region of Antioquia—Colombia, located at an altitude of 2350 m, with environmental temperatures ranging from 7 to 22 °C (average, 15 °C) and a relative humidity of 70%.

### Litters

Experimental replications were done using ten commercial crossbreed litters (Camborough 29/maternal-line × PIC 410/paternal-line), having 10 to 12 piglets each. Pigs were 7 to 9 week old, with a low weight variance (6.6 ± 0.42 kg) and were balanced for sex.

### Facilities

Evaluations were done during the nursery phase. On average, weaning was done at 28 d; thereafter, pigs were placed in nursery facilities and housed in 3 × 2.5 m pens that had a floor slightly raised of plastic slats and metal bar-walls in between. Each pen was equipped with two nipple drinkers and a hopper feeder. Feed and water were continuously available for ad libitum intake. Lights were on from 7:00 to 16:00 and the ambient temperature was ~ 25 °C.

### Musical pieces

For this research, 39 instrumental electronic music pieces (each 1 to 5 min long) were composed and produced. For the composition process, all musical pieces followed specific directives:

1. Consider pigs’ auditory perceptive characteristics (pig hearing range, 40.5 Hz to 40 kHz).
2. Maintain intra-work homogeneity in spectral or temporal acoustic parameters (e.g., low pulse content or high frequency) throughout the duration of the musical piece.
3. Produce works with homogeneity in the amount of acoustic and perceptual information over the entire duration.

Virtual Studio Technology (VST) music production techniques were applied, using computers, computer music tools, virtual instruments, and MIDI controllers. Initially, recordings were made in MIDI format with the Ableton Live 10^®^ suite from an Ableton Push 2 controller and a Fishman Triple play MIDI controller device attached to an electric guitar. Sibelius Ultimate^®^ software (AVID 2022) was used to write scores. Subsequently, the Kontakt 6 virtual instrument library with plugins for native instruments was used. No equalizers, compressors, or spatial effects were included.

Each musical piece had differentiable acoustic attributes, assessed through quantitative computer analysis, using Sonic Visualizer^®^ software (2018, Chris Cannam and Queen Mary, University of London), yielding numerical data (see Table 1). This method followed approaches used in other studies^17^. Data obtained were stored in a matrix for further analyses. Definitions were based on previous literature^28,29^ and used the following parameters:

- Centroid: center of mass of the sound spectrum; related to sound brightness and timbre
- Amplitude: distance between the peak of the wave and its base, in decibels (dB); as the wave amplitude increases, dB increase, reflecting an intensification of volume
- Dissonance: sensory dissonance measures the perceptual roughness of the sound and is based on the roughness of its spectral peaks. Given the spectral peaks, the algorithm estimates the total dissonance by summing the normalized dissonance values for each pair of peaks. These values are calculated using dissonance curves that define the dissonance between two spectral peaks as a function of their frequency and amplitude ratios.
- High frequency content (HFC) of a sound spectrum
- Zero crossings rate (ZCR): this measures the number of times the value of the signal (audio wave) crosses the zero axis. This value was usually small for periodic sounds and large for noisy sounds.
- Pulse in beats per minute (BPM); the unit of measure for the tempo-rhythm or speed
- Spectral deviation: a measure of the standard frequency deviation around the spectral centroid, indicating how much frequencies in a spectrum can deviate from the center of gravity.
- Instrumentation: number of instruments simultaneously presented. The number of instruments was always constant over the entire duration of a piece.

**Table 1:** Summary statistics of acoustic parameters of the 39 musical pieces.

Acoustic parameter	Mean	Minimum	Maximum	SD
Amplitude	0.07	0.01	0.22	0.05
Centroid	1.548	746	5.090	820
Dissonance	0.2	0.09	0.43	0.05
HFC	123.849	2.216	387.374	112.432
ZCR	0.04	0.02	0.19	0.03
BPM	114	80	160	21
Spectral deviation	2.569	1.589	5.556	854
Instrumentation	4	1	8	2

*HFC* high frequency content (kHz), *ZCR* zero crossings rate, *BPM* beats per minute, *SD* standard deviation.

## Experimental design

**Figure 1:**
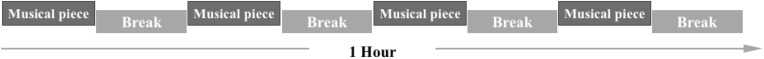
Musical stimulation arrangement. Musical pieces were randomly presented, considering interspersed rest periods.

A Bose SoundLink Air Digital loudspeaker was installed in the study location. Pigs spent at least 1 wk in the nursery facilities before musical exposition. Ten replicates were done. Each replicate (with a different litter of pigs) was performed only once, and pigs had never been stimulated with music, to avoid habituation bias. Each replicate was exposed to a musical stimulation arrangement (Fig. 1). This included four to six musical pieces randomly presented (5 stimuli per replicate, on average) with a 3-min interval without music as a break. Some pieces were presented up to twice (but not in the same litter), obtaining a total of 50 video observations of pigs reacting to a piece of music. The maximum duration of each replicate was 1 h and it was always conducted between 9:00 and 10:00 a.m. Music was played at 70 dB.

### Evaluation of emotional responses

During the stimulation arrangement, pigs’ behaviors were captured with a high-definition camera (Panasonic HC-X900. Panasonic Corporation, Hamburg, Germany), installed in front of the pen, enabling all individuals to be clearly observed. Videos were used to evaluate pigs’ emotional responses to various musical pieces, using the Qualitative Behavior Assessment (QBA), a method successfully used for evaluating emotions in several species, including horses ^30^, pigs ^15,31^, buffalos^32^, sheep^33^, dogs^34^, and elephants ^35^. This method is mainly used to evaluate animals’ emotions by integrating their body language information. It captures how individuals interact with their environment by recording “how the animal behaves” instead of “what the animal does.” ^36^. All QBA terms were initially assessed. However, scores were only obtained for 17 terms (active, agitated, bored, calm, content, fearful, friendly, happy, indifferent, inquisitive, irritable, lively, playful, positively occupied, uneasy, relaxed, and sociable) during the observations. Consequently, the terms distressed, apathetic, and frustrated were eliminated from further analyses. Each term was quantified along a 125 mm visual analog scale that indicated the intensity of each behavioral expression. Thereafter, distances (in mm) from the left margin (minimum) up to the observer's mark for each adjective were measured. Emotional responses were assessed on the entire litter. Video analysis was blind to the observer, excerpts were evaluated in randomized order and without sound. Each video evaluation session lasted 2 h and was performed within an interval of 4 d. Data obtained were collected in a matrix for statistical analyses.

### Intra and interobserver reliability

The rating was conducted by one trained observer, who performed a test–retest reliability evaluation using a sample of 10 video excerpts, randomly selected, of pigs’ litters (average of 30 s each). The intraobserver reliability for each QBA term was evaluated with Pearson's correlation coefficient, obtaining high values (r ≥ 0.90) for active, agitated, calm, content, fearful, friendly, inquisitive, happy, lively, playful and sociable; moderate (0.50 ≥ r < 0.80) for relaxed, indifferent, irritable, positively occupied and uneasy; and low (r < 0.50) for the term bored. The terms apathetic, distressed, frustrated were not identified in any of the evaluations, obtaining a score of "0;" therefore, their correlation coefficients were not analyzed. The same sample was used to perform an inter-observer reliability test between two trained observers, and Pearson’s correlation (r > 0.86) indicated excellent agreements on their scores.

### Statistical analyses

The QBA data were initially evaluated through descriptive analyses and thereafter by applying principal component analysis (PCA). This technique identifies variables underlying association patterns, allowing conformation of emotional indexes [principal components (PC)]. Subsequently, the relationship of these indexes with acoustic parameters was evaluated. Spearman's correlation coefficient was used for non-normally distributed data to describe the direction and magnitude of linear associations between acoustic parameters (predictor variables) and response variables (emotional indexes). Inferential methods were used to evaluate the statistical significance of these correlations. Given that the relationships exhibited between some acoustic parameters and the emotional indexes were evidently non-linear, a Generalized Additive Models (GAM)^37^ was used, since it allows inclusion of nonlinear relationships to the model, through replacement of the linear form $\sum {\beta }_{j}{X}_{j}$ using the sum of smoothed basis functions $\sum {S}_{j}\left({X}_{j}\right)$. GAM models were compared through the Akaike information criterion (AIC), choosing the model with the lowest value. We also tested additional tree-based methods such as Decision Trees (DT)^38^, Random Forests (RF)^39^, and XGBoost (XGB)^40^ to explore the data set. These three approaches enabled estimating nonlinear relationships and inferring the importance of predictors on the response variable. The methodological framework adopted for the training, validation and comparison of the tree-based models was oriented under a predictive approach, where stages of preprocessing, hyperparameter tuning and predictive quality assessment of the models were conceived as a single operational flow, executed with repeated cross-validation strategies to evaluate the consistency of the models over various resamples or pseudosamples. Repeated k-fold cross-validation was used with K = 10 and 5 repeats with a training set proportion of 80% and 20% for the test set, with stratified sampling on the response variable. We compared the error metric implemented to monitor training, and the root mean squared error (RMSE) was used to select each model. This metric was used in the training set and the test set; as an additional performance measure, the correlation between predicted and actual values of the test set was obtained. Finally, we compared the selected GAM model with the selected RF, DT, XGB models for each index, using the RMSE metric and Spearman correlation with each index, to select the model with best performance. All analyses were performed using R statistical software (Version 4.0.2; https://www.R-project.org) ^41^, implementing multiple libraries, including FactoMineR, tidyverse, tidymodels, DALEX, splines, mgcv, vip, among others. P ≤ 0.05 was chosen as the limit for statistical significance and tendencies were discussed for 0.05 < P < 0.10.

## Results

### Evaluation of emotional responses

Descriptive analysis of QBA terms indicated higher means for active and agitated, and lower for bored, indifferent, and irritable (Table 2). Ratings of 0 were obtained for terms distressed, apathetic, and frustrated, and consequently excluded from subsequent analyses.

**Table 2:** Summary statistics of pigs’ QBA terms (in cm; 50 video observations).

Emotional state	Mean ± SD	Min	Max	CV
Active	7.22 ± 3.55	0.5	12.5	49.17
Agitated	3.52 ± 4.35	0	12.5	123.58
Relaxed	3.04 ± 3	0	11	98.68
Fearful	3.0 ± 4.16	0	12.5	138.67
Calm	2.83 ± 3.09	0	12	109.19
Content	2.77 ± 3.13	0	9.5	113
Indifferent	0.48 ± 1.55	0	8	322.92
Friendly	2.28 ± 3.01	0	10.5	132.02
Bored	0.02 ± 0.13	0	0.9	650
Playful	2.5 ± 3.23	0	11.5	129.2
Positively occupied	2.64 ± 2.7	0	11.5	102.27
Lively	3.13 ± 3.28	0	12.5	104.79
Inquisitive	3.06 ± 3.32	0	10.5	108.5
Irritable	0.5 ± 1	0	4.8	200
Uneasy	2.92 ± 3.88	0	12.5	132.88
Sociable	2.32 ± 2.87	0	11	123.71
Happy	2.16 ± 2.86	0	12	132.41
Distressed	0	0	0	0
Apathetic	0	0	0	0
Frustrated	0	0	0	0

**Figure 2:**
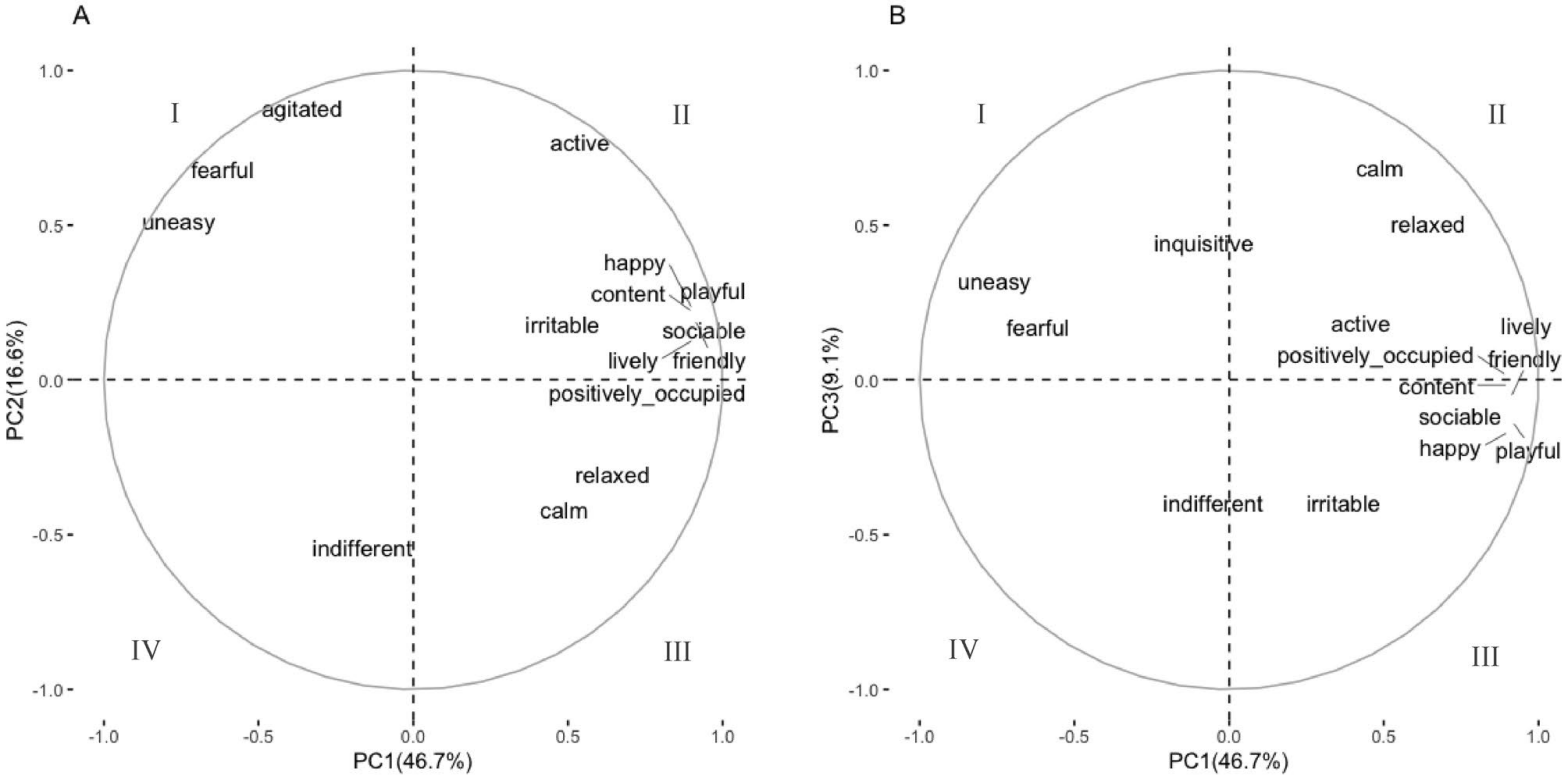
Plots of loadings for the 17 QBA analyzed terms. (**A**) Terms plotted on the first principal components PC1 (positive high arousal index) and PC2 (negative high arousal index). (**B**) Terms plotted on PC1 and PC3 (positive low arousal index).

The PCA generated three PCs with eigenvalues exceeding 1.5. Any QBA term with loading > 0.6 was considered a major contributor to each PC. PC1 explained 46.68% of the variance, and included terms content, friendly, playful, positively occupied, lively, sociable, and happy with positive loadings, and fearful and uneasy with negative loadings; this PC was characterized as positive high arousal index. PC2 (explaining 16.64% of the variance) had highest positive contributions for terms active, fearful, agitated, and uneasy, and was considered as negative high arousal index. PC3 (with 9.08% of the remaining variance) had terms relaxed and calm, and was defined as positive low arousal index (Table 3). Loading plots for PC1 and PC2, and for PC1 and PC3 are presented in Fig. 2A,B, respectively.

**Table 3:** Principal component analysis of QBA. Terms with loadings > 0.6 are bolded and were used to define the indexes identified in the analysis.

Terms	PC1 positive high arousal index	PC2 negative high arousal index	PC3 positive low arousal index
Active	0.48	**0.71**	0.24
Relaxed	0.49	**−**0.36	**0.66**
Fearful	**−0.67**	**0.62**	0.23
Agitated	**−**0.42	**0.82**	0.09
Calm	0.54	**−**0.36	**0.63**
Content	**0.90**	0.22	**−**0.02
Indifferent	**−**0.11	**−**0.49	**−**0.45
Friendly	**0.91**	0.19	**−**0.06
Bored	**−**0.03	**−**0.19	0.06
Playful	**0.92**	0.23	**−**0.13
Positively occupied	**0.90**	0.02	0.01
Lively	**0.91**	0.13	0.12
Inquisitive	**−**0.02	**−**0.37	0.50
Irritable	0.43	0.12	**−**0.34
Uneasy	**−0.70**	**0.67**	0.26
Sociable	**0.91**	0.19	**−**0.09
Happy	**0.90**	0.23	**−**0.17
Eigenvalues	7.94	2.83	1.54
Cumulative percentage of variance	46.68	63.33	72.41

**Figure 3:**
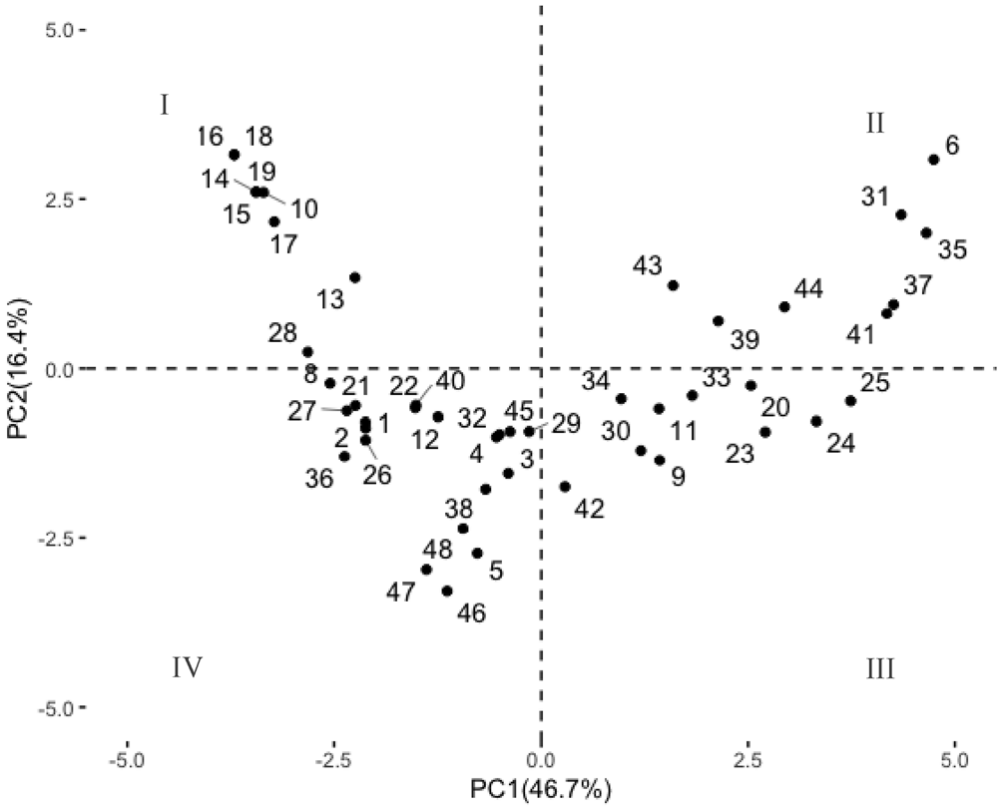
Coordinates of litter emotional states to each piece of music.

Musical pieces can be related to emotional responses according to their location in the plot. For example, quadrant II included pieces 6, 31, 35, 43, 44, 39, 37 and 41 (Fig. 3). This quadrant corresponds to positive high arousal emotional states, with QBA terms such as playful, happy, and content (Fig. 2A). In contrast, pieces located in quadrant I (i.e., 10, 13, 14, 15, 16, 17, 18, 19; Fig. 3) were related with negative emotional responses, including uneasy and fearful (Fig. 2A). Figure 3 display the spatial distribution of musical pieces (identified with codes) generated by PCA.

### Relation of acoustic parameters with the emotional index

Analyses involved various statistical methods, seeking the best technique to evaluate relationships among acoustic parameters and emotional indexes.

#### Linear modeling

In preliminary analyses, simple correlations between acoustic parameters and emotional indexes were not significant (P > 0.05). Based on the lack of linear association between evaluated variables, we inferred that the increase in one parameter did not simply imply an increase in a specific emotion. Consequently, nonlinear analyses were subsequently used.

#### Nonlinear modeling

For each emotional index, the AIC criterion was applied to select the best GAM model, taking into account the minimum AIC value generated and testing all possible structures (including different combinations of acoustic parameters). Based on that, acoustic parameters pulse and instrumentation were considered predictor variables for the positive high arousal index (P < 0.0001), explaining 46.9% of the deviance. Positive low arousal index, BPM and instrumentation were associated (P < 0.0001), explaining 40.4% of the deviance. For negative emotional index, explaining 26.3% of deviance, parameters HFC, ZCR, spectral deviation, pulse, and instrumentation were predictor variables; however, only HFC and spectral deviation had a tendency of association (P < 0.1) to this index. A summary of GAM analyses, with acoustic parameters that were predictors (based on comparisons of the additive model system) for each emotional index, and significance of the associations, is in Table 4.

**Table 4:** Association of acoustic parameters predictors for each emotional index, based on a generalized additive model (GAM).

Emotional Index	Acoustic parameters							
	Centroid	Amplitude	Dissonance	HFC	ZCR	Spectral deviation	Pulse (BPM)	Instrumentation
Positive high arousal index	NP	NP	NP	NP	NP	NP	< 0.001	< 0.001
Negative high index	NP	NP	NP	0.075	> 0.1	0.077	> 0.1	> 0.1
Positive low emotions index	NP	NP	NP	NP	NP	NP	< 0.05	< 0.001

*NP* parameters considered no predictors for model selected, *HFC* high frequency content, *ZCR* zero crossings rate, *BPM* beats per minute.

**Figure 4:**
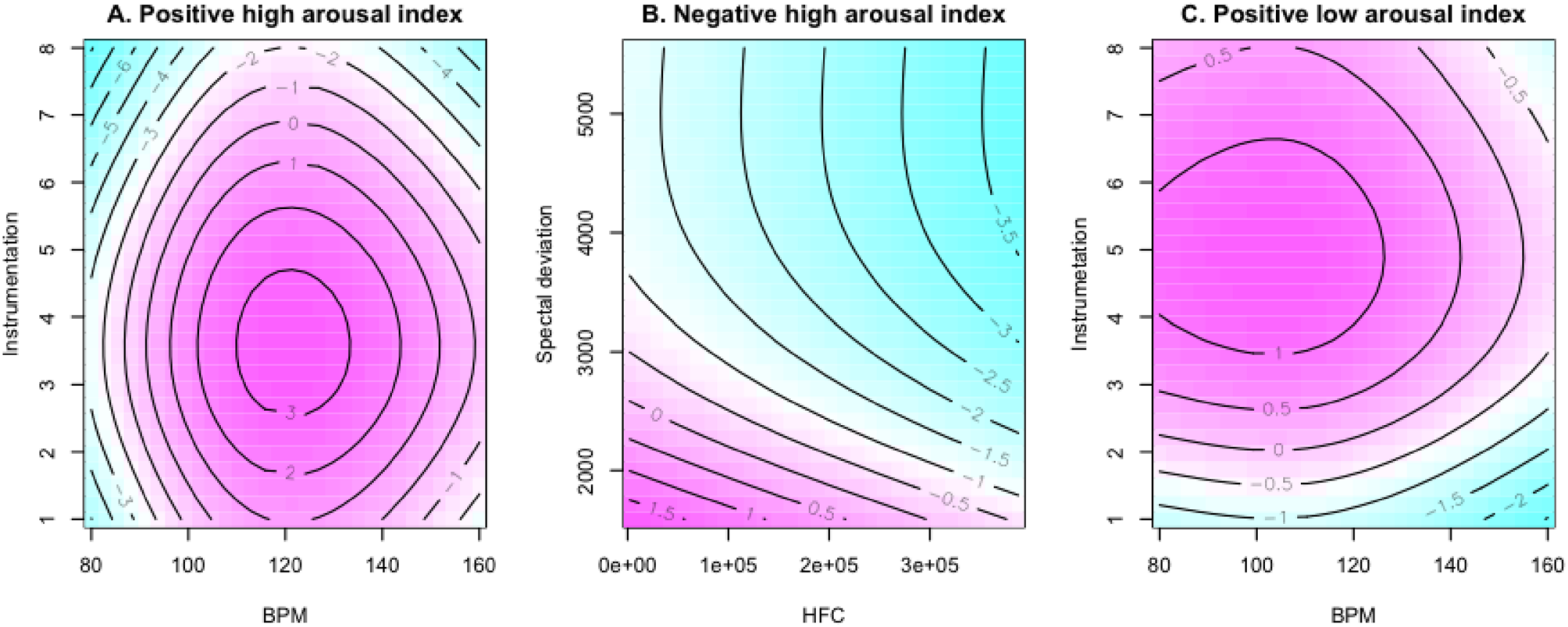
Contour plots for predictive acoustic parameters, derived from a GAM model. The central pink area determines explicit ranges where specific acoustic parameters were related to each emotional response (**A**). Positive high arousal index. Pulse values between 110 and 130 BPM, with 3 and 4 instruments, induced more positive responses. (**B**) Negative high arousal index. A combination of any HFC values, with levels of spectral deviation < 3000 were associated with higher values for this index. (**C**) Positive low arousal index. Pulse values < 120 bpm and 4 to 6 instruments were associated with emotional responses included in this index.

Contour GAM plots are in Fig. 4. Only statistically significant acoustic parameters for emotional indexes predictors were included, and the central pink area determines explicit ranges where specific acoustic parameters were related to each emotional response. Relationships between BPM and instrumentation, both predictors of positive high arousal index, are in Fig. 4A; pulse values between 110 and 130 BPM, with three or four instruments, highly influenced this index. Associations between HFC and spectral deviation, both predictors of negative high arousal index, are in Fig. 4B. A combination of any HFC values, with levels of spectral deviation < 3000, were associated with higher values for this index. Pulse and instrumentation acoustic parameters, predictors for positive low arousal index, are in Fig. 4C. Pulse values < 120 bpm, with four to six instruments simultaneously presented, had a positive association with this index.

As a complementary analysis, another methodological framework based on predictive modeling was proposed, using cross-validation strategies that generates pseudo-samples to overcome the sample size limitation of the data set. In this way, a modeling method using decision trees, Random Forests and XGBoost were applied. For each method, the selection of the best model was carried out using RMSE (Root Mean Square Error). This metric was also used to compare performance of these models with GAM (Table 5).

**Table 5:** Performance of nonlinear modelling methods comparisons and correlation between actual and predicted values on test and training sets.

Emotional Index	Model	RMSE train	RMSE test	Correlation
Positive high arousal index	GAM	2.10	2.33	0.63
	RF	2.26	2.78	0.12
	DT	2.87	3.80	0.08
	XGB	2.59	2.66	0.24
Negative high index	GAM	1.25	1.44	0.50
	RF	1.52	1.31	0.60
	DT	1.68	1.57	0.11
	XGB	1.66	1.65	0.07
Positive low emotions index	GAM	0.95	1.08	0.56
	RF	0.97	1.23	0.32
	DT	1.07	1.60	0.31
	XGB	1.04	1.22	-0.10

*RMSE* root mean square error, *GAM* generalized additive model, *RF* random forests model, *DT* decision trees model, *XGB* eXtreme gradient boosting model.

In general, the GAM has a lower value for RMSE on the test set and, therefore, better performance in capturing with more sensitively the behavioral pattern of the data, specifically in the positive high and low arousal indexes, consistent with a higher correlation with each (0.63 and 0.56 respectively). In contrast, for the negative high arousal index, the RF model had a lower RMSE and a correlation of 0.60. However, for this index the models exhibited overfitting, except for the GAM model. Therefore, we decided to discuss the results of non-linear modeling for the GAM model only.

## Discussion

To our knowledge, this is the first study evaluating relationships among several acoustic characteristics of music (using a quantitative approach) with emotional responses in non-human animals. Moreover, this is the first study considering simultaneous interactions among these parameters. Due to the lack of antecedents in the field and according to the nature of these data, several statistical approaches were used, including linear (correlation) and non-linear modeling (GAM, RF, XGB and decision trees methods). All proposed analyses aimed an exploratory working route for understanding music and emotions, inherently complex phenomena. We concluded that acoustic temporal and spectral elements of music interacted in an integrated way to modulate emotions in pigs.

Based on the QBA, musical pieces used herein generated a wide range of emotional responses, from happy and relaxed, to fearful and irritable responses in pigs, similar to our previous study on the effects of music on pigs^15^. This effect was similar to that observed in humans, with music inducing great variation in emotional responses, from sadness to excitement, anger or fear, and more^42,43^, consistent with music being regarded as one of the best-known forms of emotional communication^44,45^. Applying the PCA to QBA data grouped emotional responses into positive high, positive low, and negative high arousal indexes, according to their valences. These emotional responses were modulated by the integrated influence of the acoustic parameters in the stimuli used. Therefore, musical composition and structure were decisive in emotional responses to music in pigs, and may constitute an approximation to the knowledge we have about structural effects of music in humans^15,46,47^.

Linear associations between acoustic parameters and emotional index (the initial exploratory approach), did not explain the complex relationships among evaluated variables. Therefore, an inferential approach using GAM models and predictive modeling (RF, DT and XGB models) was explored. The GAM model is considered a method of medium complexity, which supports non-linear relationships using the lower AIC for appropriate model selection. Conversely, the RMSE metric was applied for the predictive methods to identify the best performance for the data analyzed. GAM models provided the best performance, capturing the global pattern of the data behavior identifying the association of musical parameter evaluated. Conversely, other modelling did not perform as well as we expected (presenting higher RMSE and lower correlation with each index). This was a limitation in our study, due to the limited number of musical pieces evaluated; consequently, further studies should be conducted with a larger sample size to identify the influence of more musical attributes on emotional indexes. However, this limitation does not invalidate the results and importance of this study, because this is a first step to understanding the complexity of this phenomenon.

GAM demonstrated that emotional responses observed in pigs were not explained by a single acoustic variable with direct association. This revealed the complex and simultaneous interactions among several acoustic parameters to induce a specific emotional response. Particularly, pulse and instrumentation were identified as predictor variables; musical pieces with a range of pulse between 110 and 130, with two to four instruments generated higher positive high arousal responses. Conversely, if pulse value was < 120 bpm, with four to size instruments, emotional responses were positive with low arousal.

It was reported that fast-pulse music was more effective in influencing pig behavior than slow-pulse music^21^. In the present study although certain ranges of pulse influenced emotional responses, it was not the only acoustic variable, but also instrumentation. In humans, several theorists have established connections between the information content of a piece, often discussed in terms of predictability and listener interest. The higher the information density (in number of instruments, and harmonic complexity), the longer is the temporal experience and higher effort (in terms of processing activity) is required; it may condition listener's interest^48^. Conversely, a predictable piece (less information) can be easily embedded and fragmented in the mind and, therefore, requires less processing time and less effort from the listener, which can lead to greater interest^49,50^. Therefore, the number of instruments is associated with the amount of musical information and can explain the low arousal positive emotional responses to pieces with many instruments (4–8), as more information can reduce interest and attention. When compositions were based on more simple patterns for humans, and probably for pigs, there were high arousal positive emotional responses. Consequently, emotional responses in animals will be influenced by more than one musical structural component; therefore, analyses of this type of stimulus must simultaneously include evaluation of several acoustic parameters.

We discovered that parameters like spectral deviation and HFC must also be important for the modulation of pigs’ emotional responses to music. These parameters are considered timbral attributes^51–53^. Timbre, one of the most important aspects of musical sounds^54^ is closely related to music emotions^55,56^. Although timbre is a multidimensional feature and, in turn, has other elements such as attack time, decay time, among others^51^, evaluation of several timbral attributes allows a relevant approach to this important feature. We inferred the relevance of timbre in modulation of emotional responses in pigs, with some of its attributes associated with positive high and low arousal indexes. This was consistent with human research that related timbre to emotional dimensions valence and arousal, measures of how positive and energetic music sounds^57^.

Our results specifically related to high-frequency content can be explained by the vocalization frame of the pigs. Communication has various spectro-temporal attributes encoding different information categories, and the frequency and its fluctuations provide semantic information. Piglets that were restrained and castrated produced higher high-frequency calls (also more dissonant), than control piglets that were restrained but not castrated, suggesting that high-frequency vocalizations reflected pain during castration^58^. This same framework was extrapolated to music; therefore, pieces with high dissonance and HFC can be relevant in modulation of negative emotional responses. There is no precedent for evaluating the high-frequency content of music on behavioral or emotional responses of animals. However, previous research on pigs demonstrated the importance of high frequency content in vocalizations. The study indicated that vocalizations of domestic pigs can be distinguished into high frequency calls (yelps, squeals) and low frequency calls (grunts), with two or three less distinct subcategories within each of the two main types^59^. High-frequency calls are associated to a negative context and an indicator of negative affective valence^60^. From our results, we inferred that high-frequency content in music, similarly to vocalizations, may evoke aversive experiences in pigs. However, more study is required on this hypothesis in animals, in particular on how frequency modulation interacts with and contributes to the induction of emotional responses in pigs.

In humans, the association between music and emotions derives from neurocognitive process, with music structure as determinant ^7,8,61,62^. Acoustic stimulation activates a multidimensional process in the brain. Once it is translated into neuronal activity, widely distributed brain areas participate in the neuronal encoding of music^63^. Acoustic aspects and musical structure such as rhythm, tone, melody, and harmony are processed in the frontal, temporal, and parietal regions^8,11,64,65^. The amygdala, ventral striatum, hippocampus, hypothalamus, and interaction with arousal control systems, based on norepinephrine and serotonin concentrations, have effects on emotional responses and the autonomic nervous system, inducing behavioral and organic responses^7,62,66,67^. Our results, evidencing effects of musical structure on pig emotional responses, led us to hypothesize that, in this species, music is the object of a neurocognitive process similar to humans. However, additional evaluation techniques, e.g., neuroimaging, are needed to corroborate it. Nevertheless, delving into these aspects from animal models may be relevant to understanding the neurocognitive basis of music processing.

We demonstrated that analysis of acoustic parameters, in an integrated approach, was appropriate for pigs, as it is for humans. Research in humans demonstrated that certain acoustic features were associated with specific emotional valences. For example, sadness with slow tempos, narrow frequency ranges, and decreases in tone; anger with an increase in fundamental frequency and at a higher intensity (amplitude); and fear with an increase in fundamental frequency, HFC, and a faster articulation rate^43^. These descriptions have been validated in various human studies^68,69^ and our results demonstrated that this type of characterization can be proposed in non-human animals. This provides critical knowledge for creation of species-specific acoustic sensory stimuli and demonstrates the enormous potential for environmental enrichment for animals.

Our study highlighted the importance of the psychoacoustic study of music, which to our knowledge has not been explored in nonhuman animals. Future studies should separately address testing the specific effect of each of the spectral and temporal characteristics of the stimuli while keeping other acoustic parameters stable, as well as the interaction of basic musical structural aspects with various brain features (e.g., structure, chemistry, and physiological pathways) that may elucidate the mechanisms through which music can induce specific emotional responses in nonhuman species.

## Conclusion

Emotional states in pigs are modulated by structural characteristics of music. Modulations of pulse and instrumentation were the main acoustic parameters associated with emotional responses in pigs, at least for the ranges of acoustic parameters used in this study. Our data and analyses are a starting point to design and refine acoustic sensory stimuli appropriate for environmental enrichment, with predictable and validated emotional effects.

## Data Availability

The datasets generated during the current study and code implemented for its analysis are available at https://github.com/Julianazapata/Nature-Scientific-Reports-Spectro-temporal-analysis.
